# Highlights of the eleventh annual meeting of the National Cancer Research Institute, 1–4 November 2015, Liverpool, UK

**DOI:** 10.3332/ecancer.2016.615

**Published:** 2016-01-21

**Authors:** Audrey Nailor, Ian Lewis

**Affiliations:** 1 Cancer Intelligence, 154 Cheltenham Road, Bristol, UK B56 5RL; 2 Department of Research, Tenovus, 9th Floor Gleider House, Ty Glas Road, Cardiff CF14 5BD, UK

**Keywords:** biomarkers, immunotherapy, prevention, e-cigarettes, big data

## Abstract

The annual meeting of the National Cancer Research Institute (NCRI), held in Liverpool, UK, has a solid reputation of being a multidisciplinary conference. It brings the diverse cancer interests of the United Kingdom together, from funders to researchers to clinicians. Key themes for the coming year’s innovation emerge. At this meeting, particularly notable topics were immunotherapy and prevention, with sessions on Big Data and e-cigarettes generating significant interest and discussion. Broad themes included discussions around cancer evolution, and the economic challenges of the United Kingdom’s cancer burden.

## Introduction

The annual meeting of the National Cancer Research Institute (NCRI) has always served multiple purposes; both in reflecting on the year’s past achievements and preparing for the challenges ahead.

There were over 150 speakers at the event and over 2000 estimated attendees. For the first time, professional development workshops were offered, and a new role of social media was discovered and emphasised. The addition of an app was meant to connect people online, as was the prize for the best use of the #NCRI2015 hashtag. Live tweets during the sessions garnered much attention.

‘At least a third of all cancer cases are preventable, so finding ways to encourage healthy behaviour will help prevent many cancers and other chronic diseases’, explained Dr Karen Kennedy, director of the NCRI, in an editorial supplement supplied at the beginning of the conference. ‘We also know that the earlier cancer is diagnosed, the more effective treatment will be, which is why screening and public awareness are key areas’.

‘Colleagues across the research spectrum, including patients and carers, will come together to strengthen partnership’, she added in the opening address of the conference. ‘It’s all about bringing people together to progress research’.

The NCRI brings together a wide range of funders and by joining up this diverse set of stakeholders we can help support research projects that are too complex for any one funder to take on individually.

## Evolutionary perspectives and the arms race of cancer resistance

The CR UK Lifetime Achievement Award is always a highlight of NCRI. This year Professor Mel Greaves of the Institute of Cancer Research, Sutton, UK presented a talk on Evolutionary Tales in Leukaemia [1]. Prof Greaves’, a previous year’s recipients of this award, talk emphasised the importance of the mentors who nurtured his early career, the collaborators with whom he shared his achievements, and the students and postdocs whom he developed. He also pointed out the vital role that patients had played in his research.

He provided advice to others seeking to forge a career in science, saying they should look for opportunities wherever there are gaps in knowledge. But he also very poignantly stated that the reason for his own research into childhood leukaemia had a large emotional element, having witnessed children undergoing chemotherapy.

Prof Greaves described his ground-breaking work looking at the evolutionary reasons behind how childhood leukaemia develops and how this quest has been underpinned by a good understanding of basic biology and the natural experiments provided by patients themselves. He called childhood leukaemia an ‘evolutionary dilemma and a paradox of progress,’ and emphasised the importance of an evolutionary perspective in medical philosophy.

On a smaller evolutionary scale, what can a cancer genome tell us about its past? Peter Van Loo [2] of the Francis Crick Institute, London, UK propounded a talk on the molecular archaeology of cancer and the tumour’s evolutionary history. The cancer genome can be ‘incredibly messed up,’ he said, showing a slide in which the tumour had 78 chromosomes. He compared it to a palimpsest, an ancient parchment that has been overwritten. The language is similar to evolutionary biology—an ancestral cell is the last common ancestor of the tumour, and the mutations can be mapped on a phylogenetic tree. Using NGS and bioinformatics, Van Loo explained that he can disentangle the subclonal architecture and life history of tumours from a single cancer sample.

Dr Alberto Bardelli of the University of Torino, Turin, Italy gave an excellent talk looking at the problem of resistance and tumour heterogeneity, which underpins many of the issues with targeted cancer therapies [3]. In particular he spoke about colorectal cancer and the multistep evolution of resistance to epidermal growth factor receptor (EGFR) blockade. Dr Bardelli explained that the development of resistance should not be seen as a single step ‘digital’ event, but rather a process of genetic diversification and clonal evolution. He spoke about how liquid biopsies can be used to monitor this clonal evolution and gave the example of MEK inhibition leading to a concomitant increase in KRAS. He showed that often therapies can be effective but short-lived because we are attempting to fight evolution, and trying to fight it when it is already too late. Instead we should be looking at combination therapies upfront, destroying the cell population before evolution can occur.

Moving towards the evolutionary angle of cancer resistance, Dr Charles Sawyers of Memorial Sloan Kettering Cancer Centre, New York, USA presented a plenary lecture on hormone therapy in prostate cancer [4]. He painted an eloquent picture of the arms race between treatment and acquired resistance to targeted therapy, drawing a parallel between cancer treatment and the similar behaviour of antibiotic resistance. Patterns of resistance to targeted therapy have changed, he explained. Potent inhibitors, such as the kinase inhibitors, deliver superior clinical efficacy –while driving resistance to take ever more diverse forms. As one might expect from an evolutionary model, 90% of resistance to kinase inhibitors emerge through mutation of the target protein.

The movement to next generation treatment methods has come at a price. ‘We’re starting to pay that price—opening up a Pandora’s box of diverse resistance mechanisms that are often epigenetically based’, Dr Sawyers said.

He then suggested that at cancer centres in the USA, next generation sequencing is proliferating ‘like crazy’, but that it is driven by interest in research questions, rather than patient outcomes. ‘I think we need to be better organised in how we collect information’, he said. ‘We need many answers about many things—rare somatic variants, how big an allele burden do patients need to have to be effective’. This drew an interesting parallel with other sessions on Big Data—how will all of this vast quantity of data be drawn together to improve patient outcomes?

## Reducing Europe’s cancer burden: diagnosis, economics, and policy

Dr Harpal Kumar, current CEO of Cancer Research UK, asked the question ‘Is late diagnosis of cancer real and if so what can we do about it? ‘Achieving early diagnosis has been a priority. Tweets marvelled at statistics he offered, noting the apparent disconnect between GP behaviour and cancer survival rates [5].

In 2007, Dr Kumar said, ‘We knew cancer survival in the UK was lower than in other countries,’ based on the EUROCARE-4 studies. However, the cancer community ‘didn’t know anything’ about public attitudes or beliefs about cancer. We now know that over half of all cancer diagnoses between 2006–2010 were through referrals by General Practitioners (GP), and a further 23% of cancer referrals were through the emergency service—an alarming figure which has now come down to 20%.

We now know that perceived low public awareness of cancer screening, diagnosis, and prevention does not explain our survival deficit at the international level. Dr Kumar explained the startling fact that residents of the United Kingdom are no less aware of cancer than our international counterparts, nor are we more fatalistic. However, UK residents are vastly more concerned about ‘wasting their doctor’s time’.

What does the UK need for the future? Dr Kumar says more research and action across the diagnosis pathway, including better ways to identify early stage disease, such as through biomarkers, improved screening, and greater emphasis on diagnosis. ‘CR UK will be investing heavily in this’, he said.

The highlight of the final day was the session on the Economic Impact of Cancer, chaired by BBC Economics Editor Robert Peston, who gave an incredibly frank and emotive account of the experience of his late wife’s lung cancer. His main points were that the economics of cancer are appalling with too much money spent on trying to treat the cancer at the late stages of the disease with a lack of investment in early diagnosis.

Expanding these themes beyond Britain’s borders, Prof Richard Sullivan [6] outlined some of the key and very stark challenges faced by health systems across the Europena Union (EU). Whilst the challenges of ageing populations, increasing incidence and shrinking health budgets are uniform, the spending on cancer care across each country varies massively. It is not just in relation to cancer drug spending, but also the structure and culture of care especially in radiotherapy and surgery. These differences across borders will lead to differences in care and outcomes leading to onco-migration—people moving across borders to access care unavailable in their own countries. This he noted is happening already.

Also notable was Prof Maarten Ijzerman who spoke about the value placed on different types of cancer treatment, particularly in the world of personalised medicine [7].

Finally the session was closed by an incredibly stimulating talk by Dr Tito Fojo who urged us to look beyond the published data and cast a critical eye over the claims made about cancer drugs [8]. He illustrated how increasingly marginal improvements seen with some cancer drugs are being achieved statistically by using large cohorts of patients, that doses are often reduced in patients taking toxic therapies leading to suboptimal outcomes, that in a clinical trial not everyone will achieve the median benefit of that drug, and that some might do considerably less. He stated that we are developing and approving drugs that are not only achieving very little but also may be harming a large number of patients, at an exorbitant cost—and amongst his recommendations was that we should go back to curing cancer as the goal.

## E-cigarettes are here to stay, but what are the implications?

The debate on e-cigarettes was fascinating and well-attended. This was probably one of the most interesting Q & A sessions of the conference, highlighting the fact that this is such a rapidly emerging and at times divisive issue. The healthcare professionals and researchers providing the debate and questions noted that this topic was remarkably complex.

What is clear from the data presented in this session is that there is a mismatch between the emerging evidence and the perceptions of the public. Despite the biological evidence increasingly showing that in short term they are far less harmful than cigarettes the perception that they are just as harmful has increased year on year.

Similarly, the concerns of some policymakers that e-cigarettes could lead to a gateway to smoking for children seem to be misplaced. Yet some governments across the world are seeking legislation to restrict their use or ban them outright. This is despite the fact that for many e-cigarettes are being successfully used to reduce tobacco usage or as a cessation tool.

There could be a danger that we are missing an opportunity for harm reduction for one of the biggest risk factors for developing cancer and other life-limiting diseases. This is also further complicated by the fact that EU legislation will soon mean that the only companies that may be able to afford a medical licence for the device come from the tobacco industry.

This could lead to an incredibly difficult moral quandary where health systems in the EU are only able to prescribe an e-cigarette developed and marketed by the company that you are trying to stop them from buying cigarettes.

## Perspectives on prevention

The first debate of the meeting was a hosted panel entitled ‘This house believes we should stop focusing on the causes of breast cancer and get on with strategies to prevent the disease’. Two experts on the topic sat on each side of the debate: should cancer researchers focus their attention on breast cancer causes or prevention? On the causes side sat Dr Douglas Easton of the University of Cambridge, UK and Dr Tim Key of the University of Oxford, UK; on the prevention side sat Dr Annie Anderson of the University of Dundee, UK and Dr Gareth Evans of the University of Manchester, UK.

‘We’ve got enough to start acting now’, said Dr Anderson’. We need to act on it—we can’t be wasting time with the minutiae of another wee cause’.

The experts enjoyed a healthy, respectful debate with all of them noting that prevention is frequently overlooked and underfunded despite being highly important, and that the point of the debate was not to undermine the efforts of cancer researchers to determine the causes of breast cancer. The debate was attended by a full house, who voted on their feelings before and after the debate. The NCRI encouraged attendees to weigh in on Twitter using the #NCRIBCDeb hashtag. In the end, the house motioned that it felt prevention was of more importance.

There is a growing evidence base for the importance of exercise in not only preventing cancer but also reducing the risk of it returning if you have already been successfully treated for the disease. As outlined by Prof Robert Thomas of Bedford and Addenbrooke’s Cambridge University Hospitals, UK, in the session ‘Physical Activity: the Panacea’, exercise can also help improve a whole range of quality of life issues for cancer patients, including fatigue, weight gain, hot flushes, loss of strength, peripheral neuropathy, mood, anxiety, and depression [9]. In fact, Prof Thomas stated that ‘if this was a drug, these results would be on the front page of the newspaper based on the evidence we have now’.

## Advances in cancer biology—looking forward

Dr Fabrice André of the Institut Gustav Roussy presented a talk on genomics and personalised medicine. Precision medicine aims to biopsy the tumour, perform molecular genomic profiling, identification of alterations, and providing therapy according to the biology of the tumour [10].

‘We don’t know (yet) how to interpret biology at the individual level’, he said. Trials are not designed to evaluate the efficacy of precision medicine, but to discover new drug matched to genomic alterations, and to speed up drug development.

Dr André explained that cancer drug development falls into three broad categories; drugs matched with pathway, drugs matched with alterations with low bioactivity, drugs matched with alterations with strong bioactivity. The last approach is more likely to be associated with efficacy.

How to resolve this? Dr André suggests designing trials according to target characteristics. He concludes that developing methods to identify drivers in individuals will need shared data like the GENIE project, and that trials must have three different aims—molecular screening, stratified medicine, and personalised medicine trials; that particularly in breast cancer, pathway analysis is a promising approach; and that predicting immunogenic cell death could allow personalised sequential use of drugs.

Prof Tim Hunt, of the Francis Crick Institute London, UK gave a personal insight into how cancer has touched his own life and driven his work that led to the discovery of the cyclin-dependent kinases that tell our cells how to grow and divide. Prof Hunt’s talk was a fascinating insight into career that appears underpinned by a very deep curiosity, starting with sea urchin eggs, and leading to unpicking the mechanisms that underpin cell division. Cancer after all is a matter of cell division.

Despite the huge role Prof Hunt has played in the understanding of this process, he also was quick to point out that if a surgeon can remove the cancer then ‘to hell with the molecular stuff’ and that for research to be effective it ultimately has to be translated into cancer care.

Prof Dennis Slamon of the Herceptin UCLA Jonsson Comprehensive Cancer Centre, California, USA followed this talk seamlessly by talking about taking CDK4 inhibitors into clinic. He started though by talking about the vital importance of working between the lab and the clinic, and from bench to bedside and back again.

He also talked about the molecular diversity of human cancers and noted that by only studying three or four cell lines you may not be reaching the full heterogeneity of cells seen in a tumour population. He noted his experience of developing Herceptin, a drug that has taken HER-2 positive patients from the worst outcomes for breast cancer patients to some of the best.

He spoke about the Trio Global Investigator Network which is looking at repurposing existing drugs to treat triple negative breast cancers, making the excellent point that these are not patients that failed treatment but rather women whose therapies have failed them.

At present about 45% of triple negative breast cancer patients do well and 55% very poorly—and we cannot currently predict what underlies this difference in progression-free survival (PFS). Slamon’s team have identified a drug called palbociclib that had previously been dismissed as not effective was actually able to treat advanced disease. Slamon used this as a cautionary tale for the audience, that ‘robots’ (i.e, high throughput screening) can give you answers quickly—including the wrong ones.

## The ‘fourth dimension’ in radiotherapy

Prof Uwe Oelfke of the Institute of Cancer Research, Sutton, UK started his lecture by apologising in advance that he was delivering physics so early in the morning in a German accent. His apology was not needed as this was one of the most engaging talks of the conference. It seemed most appropriate when he started his talk with a quote from the ‘Star Trek’ character Captain Jean Luc Picard. The technology and science present in his talk looked like it had come from the distant future, but this was technology that is already starting to benefit patients today [11].

Prof Oelfke gave an update on the latest developments in radiotherapy, a mode of treatment that arguably does not get the attention it deserves in the public and policy arena despite being used in the treatment of nearly half of all cancer patients.

He explained what can currently be achieved with conformal IMRT and how you can target tumours very precisely and in 3D—despite this being achieved with a series of photons shot in straight lines—and also how proton therapy allows improved dose patterns within the target.

However, he showed that there is no use having the best guns without the best targeting, and without targeting you are just trying to hit an invisible target with an invisible beam. This is further complicated by the fact that tumours are dynamic and can move not only from the time of scan to time of treatment, but can also shift during the natural breathing and heartbeat of the patient as they are being treated. Therefore, in an even more futuristic twist, he spoke about the importance of the fourth dimension: time. He noted that awareness of these dynamics will lead to the future of real-time, image-guided radiotherapy technology. He illustrated this with a CGI video of a lung tumour being treated with radiotherapy, the beam modulating in strength and direction as it moved with the heartbeat and breath of the patient.

## Narratives in Big Data

‘In order to do what I’m about to talk about, we have to bring stakeholders together from all across the industry’, explained Prof Amy Abernethy, of Flatiron Health, New York, USA as she began her lecture on Big Data [12].

The opportunities that ‘Big Data’ has for improving the care received by patients are huge. However, the type of high quality data that would be required to make real change are probably only captured from the 3% of patients that are in clinical studies. The data from those people treated without high quality data capture or surveillance represents a huge missed opportunity. Prof Abernethy talked about how this lack of systematic data capture could be addressed through new technology, particularly real time collection of patient reported outcome measures (PROMs) which could feed into an electronic health record.

What was clear from the intelligence that Prof Abernethy could glean from this data is that this information could have a huge impact on the service provided by health professionals; allowing them more time to spend with their patients, make more confident decisions, and better match the care to the individual patient.

Sadly the reality of the current situation was brought into sharp focus by a question from a carer for someone currently going through cancertreatment. This patient advocate spoke up with a powerful, emotive description of her experiences accessing cancer care in the UK, which seemed completely at odds with Prof Abernethy’s hopes for Big Data. Her experience was one of hand-written paper records riddled with errors, no communication between different parts of the service, and very little input from them as service users. This provided a truly important perspective of the gap between the high-tech advancements of the conference, and the reality of cancer patient care in the UK.

Prof Abernethy very eloquently concluded that the ‘river of data’ that could be generated by collecting real time research quality data from patients could be used to ‘power our mill’ in terms of service development and clinical improvements. Hopefully the benefits of Big Data will impact not just researchers but improve patient outcomes.

‘It’s time to bring the patient story back to the patients’, she said.

## Conclusion

Last year we noted that NCRI was engaging with social media in a new way, with the live Twitter feed and suggestion boards [13]. This year, #NCRI15 was an active hashtag on Twitter, with attendees snapping pictures of slides with smartphones and tweeting particularly pithy quotes from speakers. This added an interesting extra dimension to the coverage of the conference, and we will be excited to see how this develops in 2016.

There was a particularly poignant contrast at this meeting between the high-tech, futuristic advances that we celebrated—from e-cigarettes to the frontiers of radiotherapy—and the sobering experiences of cancer patients in the UK. Dr Kumar’s research showed that British patients ‘don’t want to bother their doctors’ to the moving story of the patient advocate, it is clear that there is work to be done to bridge the divide between the UK’s remarkable research advancements and our patient outcomes.

As director Dr Kennedy said, ‘the importance of the NCRI conference is to bring together the diverse stakeholders of cancer in the UK’. The take-home message for our correspondents showed that there is significant value to be had in this kind of cross-profession, multidisciplinary meeting—and that it grounds all of our work in the most important goal of all, that of improving cancer outcomes.
